# Molecular dynamics simulation unveils the conformational flexibility of the interdomain linker in the bacterial transcriptional regulator GabR from *Bacillus subtilis* bound to pyridoxal 5’-phosphate

**DOI:** 10.1371/journal.pone.0189270

**Published:** 2017-12-18

**Authors:** Teresa Milano, Adnan Gulzar, Daniele Narzi, Leonardo Guidoni, Stefano Pascarella

**Affiliations:** 1 Dipartimento di Scienze biochimiche “A. Rossi Fanelli”, Sapienza Università di Roma, Italy; 2 Dipartimento di Scienze Fisiche e Chimiche, Università degli Studi dell’Aquila, L’Aquila, Italy; University of California Berkeley, UNITED STATES

## Abstract

GabR from *Bacillus subtilis* is a transcriptional regulator belonging to the MocR subfamily of the GntR regulators. The structure of the MocR regulators is characterized by the presence of two domains: i) a N-terminal domain, about 60 residue long, possessing the winged-Helix-Turn-Helix (wHTH) architecture with DNA recognition and binding capability; ii) a C-terminal domain (about 350 residue) folded as the pyridoxal 5’-phosphate (PLP) dependent aspartate aminotransferase (AAT) with dimerization and effector binding functions. The two domains are linked to each other by a peptide bridge. Although structural and functional characterization of MocRs is proceeding at a fast pace, virtually nothing is know about the molecular changes induced by the effector binding and on how these modifications influence the properties of the regulator. An extensive molecular dynamics simulation on the crystallographic structure of the homodimeric *B*. *subtilis* GabR has been undertaken with the aim to envisage the role and the importance of conformational flexibility in the action of GabR. Molecular dynamics has been calculated for the apo (without PLP) and holo (with PLP bound) forms of the GabR. A comparison between the molecular dynamics trajectories calculated for the two GabR forms suggested that one of the wHTH domain detaches from the AAT-like domain in the GabR PLP-bound form. The most evident conformational change in the holo PLP-bound form is represented by the rotation and the subsequent detachment from the subunit surface of one of the wHTH domains. The movement is mediated by a rearrangement of the linker connecting the AAT domain possibly triggered by the presence of the negative charge of the PLP cofactor. This is the second most significant conformational modification. The C-terminal section of the linker docks into the “active site” pocket and establish stabilizing contacts consisting of hydrogen-bonds, salt-bridges and hydrophobic interactions.

## Introduction

The proteins of the transcription factor family named GntR, are characterized by the presence of two domains [1, 2]: the N-terminal domain, 60 residue long on average, possesses the winged-Helix-Turn-Helix architecture (wHTH) to which DNA recognition and binding [3, 4] functions are associated; the larger, C-terminal domain can belong to at least four different structural families and has oligomerization and effector binding functions. A GntR subfamily, named MocR [5, 6] after designation of the regulator of expression of rhizopine catabolism genes [7], is characterized by possessing a large C-terminal domain (350 residue on average) folded as type-I pyridoxal 5’-phosphate (PLP)-dependent enzymes [8]. Aspartate aminotransferase (AAT) [9] is the archetypal enzyme representing this fold. The two wHTH and AAT domains are linked to each other by a peptide bridge which can be of various lengths in different MocRs [10, 11].

Since their discovery, several MocR regulators have been studied and characterized. For example: TauR which activates the expression of taurine utilization genes in *Rhodobacter capsulatus* [12]; *Bacillus subtilis* GabR [13] which with PLP and γ-amino butyric acid (GABA) bound as external aldimine, activates transcription of genes coding for GABA aminotransferase and succinic semi-aldehyde dehydrogenase; PtsJ regulates the production of pyridoxal kinase in *Salmonella typhimurium* [14] while PdxR is involved in the regulation of the PLP synthesis in several bacteria such as *Corynebacterium glutamicum* [15], *Streptococcus pneumoniae* [16], *Listeria monocytogenes* [17], *Streptococcus mutans* [18], *Bacillus clausii* [19]. Recently, a new *Brevibacillus brevis* MocR has been demonstrated to regulate the expression of the gene coding for d-alanyl-d-alanine ligase [20].

The MocR subfamily contains other subgroups of regulators [21] that are predicted to regulate genes coding for different types of proteins, including membrane transporters [22, 23].

In general, the MocR-like regulators are involved as activators or repressors in the control of many, important metabolic networks yet mostly uncharacterized [23]. Despite their significance, very little is known about the molecular mechanism underlying their function and their response to effector binding. Moreover, relatively scarce structural information has been produced up to date: only the crystallographic structure of GabR is available as a starting point to envisage the mechanism with which the regulator reacts to effector binding [24, 25]. The structure revealed a swapped homodimer in which each subunit consists of a wHTH domain connected to the AAT-like fold C-terminal domain by a 29-residue linker delimited by sequence positions 81–109. Very recently, the structures of the dimeric GabR AAT domains in complex with the external aldimine formed by the PLP and the GABA have been deposited in the PDB by two competing groups [26, 27].

However, the crystallographic structures represent in general a static snapshot of the possible conformations among which the proteins may fluctuate in proximity of their free energy minimum. Moreover, crystal forces and contacts may alter the conformation of portions of the crystallized proteins [28, 29]. According to the crystallographic structure, the GabR regulator has a head-to-tail swapped homo dimeric architecture where the wHTH domain of a subunit is interacting with the other subunit. It cannot be easy to imagine on the sole basis of this static asset how the GabR regulator could respond to effector binding while changing its DNA recognition properties. Data from literature strongly support the notion that binding of PLP and GABA alters GabR conformation. For example, Belitsky reported that GabR is able to activate transcription only in the presence of PLP and GABA [13] *in vitro*. Allosteric transition is generally believed to be one of the most important mechanism through which bacterial regulators can act as molecular switches upon binding to low molecular weight effectors [30, 31]. More recently, spectroscopic, crystallographic and calorimetry experiments suggested that binding of GABA causes a conformational change altering the interaction of GabR with DNA [32, 33]. The recent availability of the crystallographic structure of the GabR AAT domains solved as a complex with the PLP-GABA external aldimine [26, 27] suggested that the presence of an external aldimine induces the transition from the open to closed conformation, typical of most of the PLP dependent enzymes of fold type-I [34]. According to the same authors, the conformational transition would trigger the activation of transcription. Moreover, several considerations [24] suggested that the linker is important for the mutual interaction of the two domains and that it should undergo a significant conformational transition upon ligand binding. Accordingly, atomic force microscopy indicated that GABA binding promotes a GabR conformational change along with a decrease in DNA binding affinity [35].

To envisage the role and the importance of conformational flexibility in the action of this bacterial transcriptional regulator, an extensive molecular dynamics (MD) study on the apo (without PLP) and holo (with PLP) forms of the GabR from *Bacillus subtilis* was undertaken. Molecular dynamics is a computational technique able to simulate the atom motions of a molecular system using a force field describing the different atom interactions and the relative forces [36]. This approach is now routinely and widely used to simulate and study the conformational changes of proteins and protein complexes as, for example, lobe and domain motions or allosteric transitions [37–39]. Molecular dynamics has already been applied to the characterization of several transcriptional regulators of different types: to cite only a few examples, MD was utilized to study the conformational flexibility of the DNA binding domain of the papillomavirus E2 transcriptional regulator [40]; the MD-guided site mutational analysis identified the ligand-binding site in HucR, the hypothetical uricase regulator of *Deinococcus radiodurans* [41]; MD simulations predicted the mode of DNA-protein interaction in the Fis regulator from *Pasteurella multocida* [42]; lastly, MD was used to test two models of allosteric mechanism of the Tet repressor [43].

A comparison between the molecular dynamics trajectories calculated for the apo and the holo, PLP bound, form of the GabR suggested that the presence of PLP may destabilize the interaction between the wHTH domain of one subunit and the AAT domain of the other. Moreover, the linker seems to play a significant role in the wHTH conformational changes in the holo GabR form. A possible linker-centered mechanism explaining the release of wHTH domain from the regulator surface is proposed and the role of a few relevant residues is hypothesized.

## Materials and methods

Multiple sequence alignment and editing was carried out with Clustal omega [44] and the software Jalview [45], respectively.

Atomic coordinates were taken from the Protein Data Bank (PDB) [46]: the apo (without PLP) and holo (with PLP) forms of the GabR from *Bacillus subtilis* are deposited and denoted by the codes 4MGR and 4N0B, respectively. The 4MGR structure is complexed with a molecule of imidazole bound at the active site of each subunit. Missing or incomplete residues in holo GabR were modeled using Modeller version 9.11 [47]. In particular, residues of the loops of the AAT domains corresponding to the sequence positions 436 to 439 and 437 to 440 in chains A and B respectively, were missing in the structure 4N0B. These loops were rebuilt using as a template the structure of the apo form (4MGR) for which the corresponding coordinates are available. Ten models were generated by Modeller and the model with the best value of the Modeller scoring function, indicating the degree of satisfaction of spatial restraints, was selected for molecular dynamics experiments. The linkers connecting the wHTH and the AAT domains were instead fully defined in both crystallographic structures chosen and therefore no rebuilding was needed.

The two models representative of the apo and holo forms, prepared as above described starting from the X-ray structures retrieved from the PDB (code 4MGR and 4N0B), have been subjected to MD simulations in water solution using the Gromacs software package [48]. The 4MGR apo form was simulated both with and without imidazole at the active sites. The simulation of the 4MGR complex with imidazole was meant to serve as a reference for validating the simulation of the imidazole-free form and detecting possible artifacts. The protein residues have been described using the AMBER99SB-ILDN force field [49]. In the case of the holo form, the PLP residue was described by the generalized Amber force field (GAFF) [50]. Partial charges of the atoms composing PLP have been calculated with the restrained electrostatic potential method [51]. Optimization and electrostatic potential analysis were performed by using Gaussian 03 [52] at the Hartree-Fock level with the 6-31G* basis set. Protonation states of titratable residues were chosen using the PROPKA web server [53, 54].

Particle Mesh Ewald (PME) method [55] was employed for the calculation of long range electrostatic interactions with a grid spacing of 0.12 nm and a short range cutoff of 1.0 nm. Bond lengths involving hydrogen atoms have been constrained to a constant value using the LINCS algorithm [56]. About 50,000 TIP3p water molecules [57] have been used to solvate the systems, imposing a minimum distance between the solute and the box of 1.4 nm. Na^+^ and Cl^−^ ions were added at physiological concentration (150 mM) with an excess of Na^+^ in order to compensate the net negative charge of the systems. The simulated systems consist of about 165,000 atoms.

Equilibrium MD simulations have been preceded by 500 steps of energy minimization using a steepest descent algorithm followed by 3 ns of MD simulation with harmonic position restraints in NVT ensemble and 3 ns of MD simulation with harmonic position restraints in NPT ensemble. Position restraints were applied on the heavy atoms of the proteins with a force constant of 1000 kJ mol^−1^ nm^−2^.

The temperature was kept constant by coupling the system to the v-rescale thermostat [58] at 298 K. Equilibrium simulations have been carried out in NPT ensemble using the Berendsen pressure bath [59] with P = 1 bar. A time step of 2 fs was used for numerical integration of the equations of motion. Summarizing, two MD simulations of the GabR protein in its Apo and Holo states have been carried out with a simulation time length of respectively 200 ns and 350 ns.

Visual and numerical trajectory analyses have been carried out with the software VMD 1.9.3 [60] and the tools of the Gromacs package [61]. Structures have been displayed and analyzed with the graphic programs Open Source PyMol v. 1.8 [62] or Chimera v. 1.11 [63]. Analysis of contacts and interactions between PLP and GabR residues and backbone was carried out with the software LigPlus [64]. Ad-hoc Perl and Python scripts were written whenever necessary.

## Results

### Structure flexibility: Linker and wHTH domain movements

This work was conceived to investigate the conformational flexibility of GabR and the influence of the pyridoxal 5’-phosphate on its dynamics properties. To ease the detection of changes induced by the presence of PLP, a comparative approach has been undertaken. Molecular dynamics trajectories have been calculated for GabR apo forms, namely GabR without any ligand (corresponding to the PDB file 4MGR after removal of imidazole), and for GabR holo form, i.e. GabR with PLP bound through a Schiff base to each subunit of the homo dimer (PDB code 4N0B).

**Fig 1:**
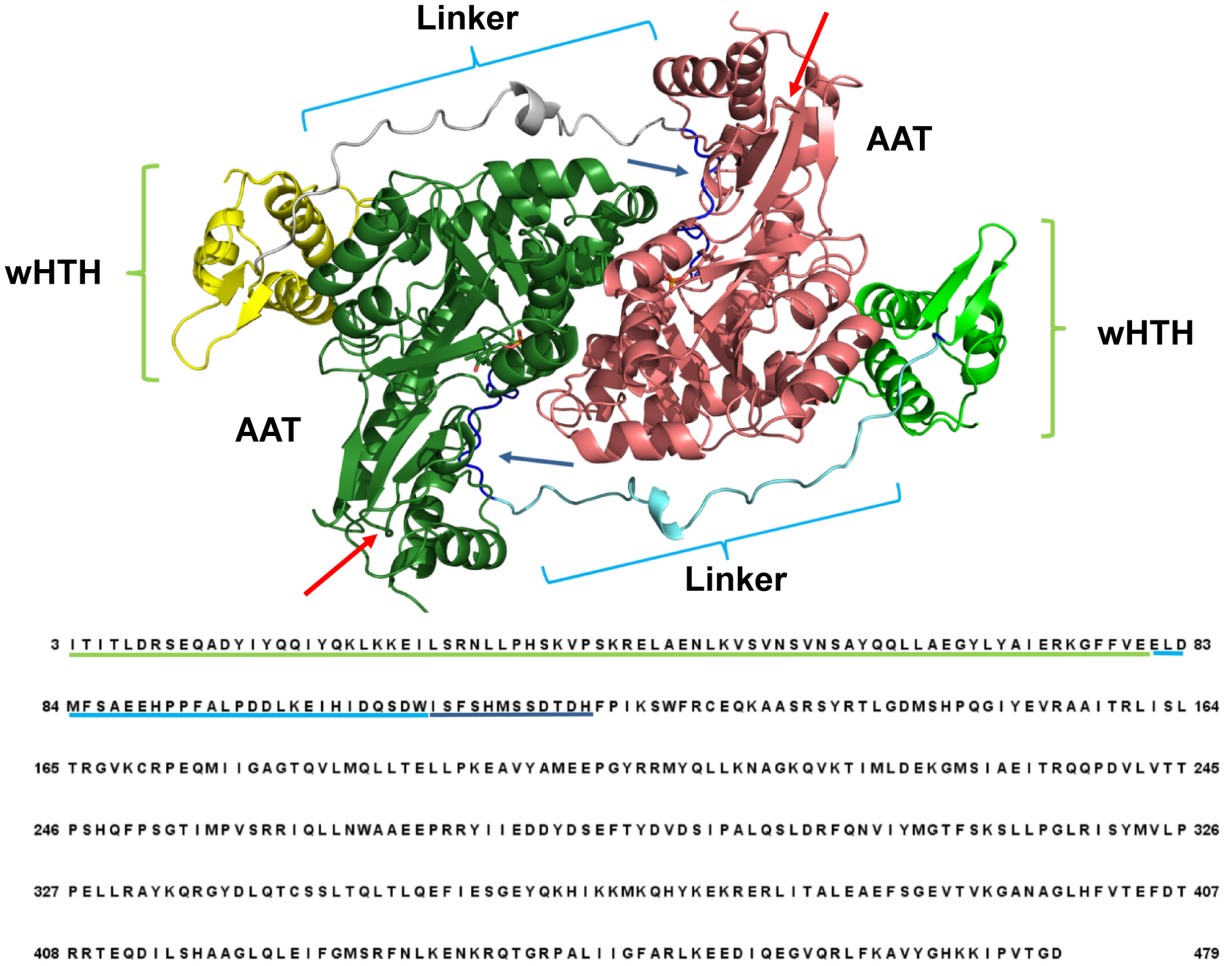
Scheme of the structure of GabR dimer with the corresponding amino acid sequence. Ribbon colors indicate the different domains in the two subunits: yellow and light green indicate the wHTH domains; cyan and grey the two linkers; dark green and dark pink the AAT domains. Green and cyan underlines map the positions of the wHTH and linker domains onto the sequence, respectively. Dark blue underlines and arrows mark the loop connected to the linker. Cyan and green curly brackets indicate respectively the location of the linkers and HTH domains on the structure. Red arrows mark the position of the two rebuilt loops. Labels ease the visual identification of the domains. Sequence numbering corresponds to that used in the manuscript text.

Fig 1 displays the structure of holo GabR based on the PDB structure 4N0B and indicates the position of the wHTH domains and linker regions on the homo dimer and on the sequence. The loop delimited by the sequence positions 110–121 that connects the linker to the first helix of the AAT domain (Fig 1) and forms part of the PLP binding site, is also indicated along with the two reconstructed short loops missing in the original crystallographic structure.

To assure that the removal of imidazole from 4MGR active site had not caused alterations of the apo GabR properties, 100 ns of molecular dynamics of 4MGR with and without imidazole have been compared. The RMSD (Root Mean Square Deviation) variations during simulation time of the imidazole and imidazole-free apo GabR forms suggests that the two structures have a very similar dynamics behavior (S1 Fig). Therefore it can be assumed that the imidazole-free apo form is not affected by any significant artifacts and it is as stable as the original structure. For this reason, it can be used as the reference apo form throughout the work.

Simulations for the apo and holo forms have been initially carried out for 200 ns. Variations of several structural and dynamics properties in the two GabR forms have been compared.

**Fig 2:**
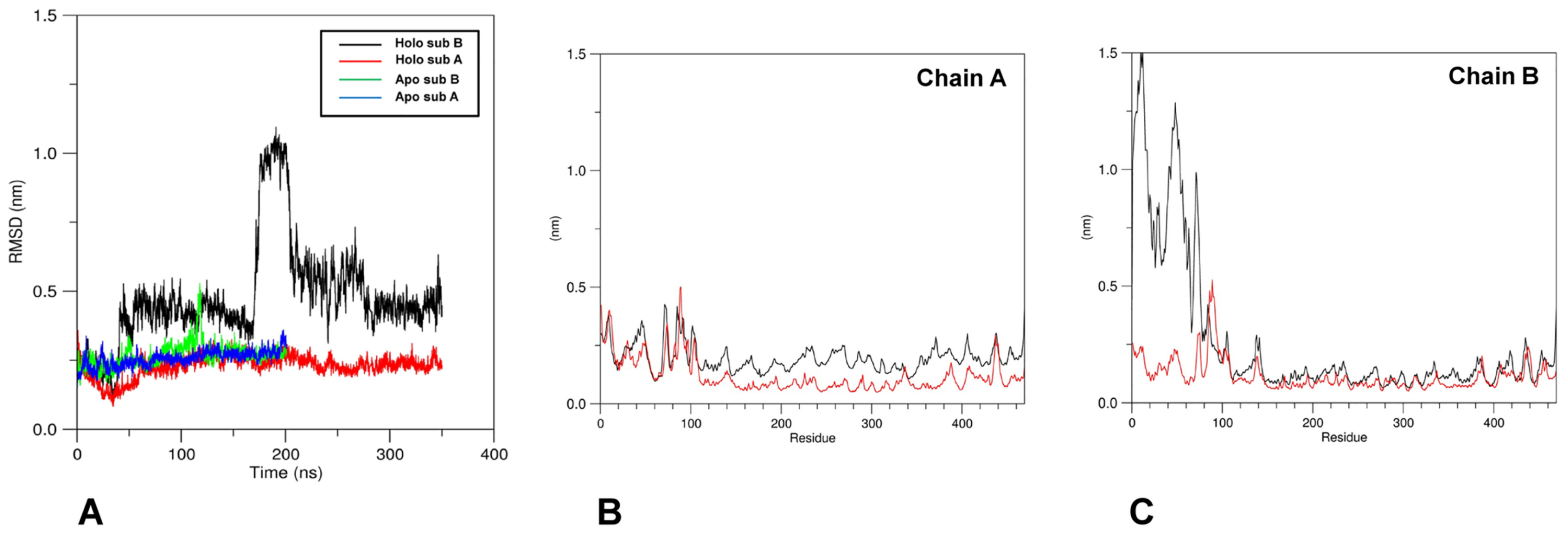
Structural variations in the subunits of holo and apo GabR forms. (A) RMSD variations. X-axis reports the time frame in ns units. Color code for attribution of lines is reported in the figure inset. All frames were superimposed to the initial reference structure. RMSF variations for (B) subunit A and (C) subunit B of both forms. All frames were superimposed to the initial structure. Black and red lines refer to holo and apo GabR, respectively. Green and cyan horizontal curly brackets indicate the sequence positions of the wHTH and linker domains, respectively. All the calculations have been carried out taking into account only the main chain atoms.

As a first assessment, visual inspection of the trajectories calculated for the two GabR forms has shown that the most evident difference found between the two structures during the simulation is represented by the movement of the wHTH domains of the holo chain B. The domain indeed tends to move farther from the other subunit (chain A) surface after about 175 ns from simulation start. On the contrary, in apo GabR, each wHTH domain remains attached to the AAT domain of the other subunit. To explore more extensively the wHTH movement, the MD simulation for the holo form was extended for further 150 ns up to a total duration of 350 ns. Fig 2A reports the plot of RMSD variations versus simulation time of each subunit of the two GabR forms with respect to the initial reference structure. The plot suggests that the GabR structures are stable under dynamics conditions while the dramatic increase of RMSD in the subunit B of holo GabR at about 175 ns is a consequence of the movement of the wHTH domain.

Analysis of the RMSF (Root Mean Square Fluctuation) reported in Fig 2B and 2C pinpoints that the increase of the RMSD in the subunit B is caused mainly by the movement of the corresponding N-terminal wHTH domain. In addition to that, the same graph indicates that the linker region is the most flexible portion in both subunits of either GabR forms. Indeed the mainchain regions approximately encompassed by sequence positions 60–100 display a few peaks in the RMSF graph which suggest wide movements around the average conformation.

**Fig 3:**
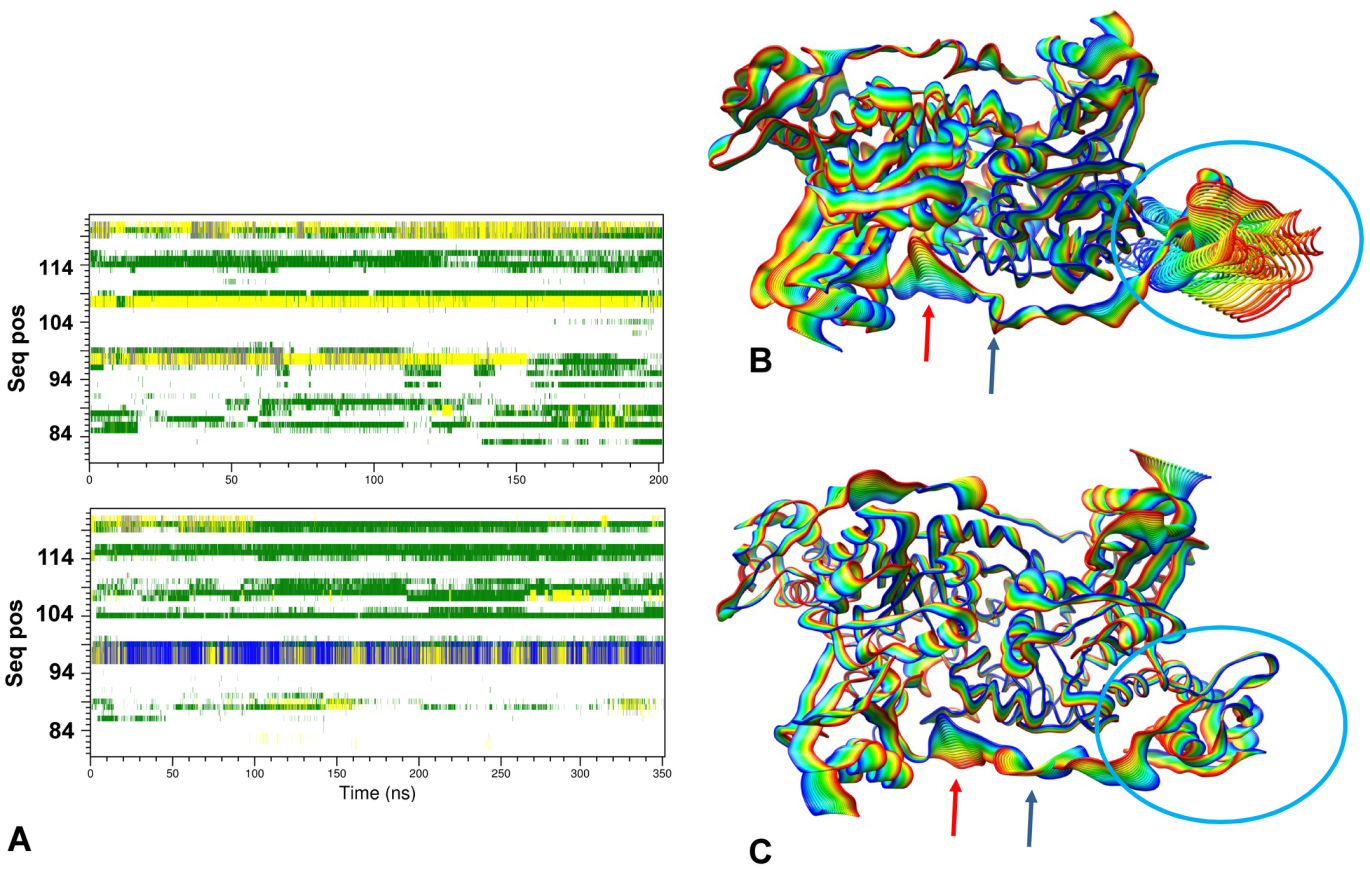
Secondary structure variations in the linker region and essential dynamics. (A) Comparison of the secondary structure variations in the linker regions of apo and holo GabR are displayed in the upper and lower subfigure, respectively. Only results for linkers of subunits B are reported. X axis reports simulation time in ns units while the Y axes indicates the sequence positions according to the numbering of Fig 1. Superposed frames of the trajectories projected onto the first essential dynamics eigenvector for (B) holo and (C) apo GabR. Frames were sampled every 17.5 and 10.0 ns, respectively. Rainbow colors ranges from blue (0 ns) to red (last frame of the simulation). Red and green arrows point to the linker loop docking into the active site cavity and the linker hinge region, respectively. Cyan circle encloses the moving wHTH domain. Reference XYZ axis are displayed in the lower right corner of each figure. The structures are oriented approximately as in Fig 1.

Moreover, analysis of the conformation variations of the linker region *versus* simulation time (Fig 3A) highlights differences in the secondary structure especially at the positions encompassed by Pro96-Leu99 where a one-turn α-helix, missing in the apo form linker, occurs (Fig 3A). All these results suggest that the role of the linker in the GabR conformational changes cannot be overlooked.

The wHTH domain of the holo chain B, after detaching from the AAT domain surface, becomes more flexible as suggested by the increase of the RMSD and of the RMSF (S2 Fig). On the contrary, the HTH domain of the holo chain A and the HTH domains of the apo GabR remain more rigid (S2 Fig).

### Dominant domain motions

The dominant motions of the holo and apo GabR were captured using the essential dynamics technique [65] as implemented in the Gromacs tools. Comparison between the movements projected on the first eigenvector of holo and apo GabR forms shows that the major differences are in correspondence of the linker and the wHTH domain of the chain B.

As far as it concerns the linker, the region encompassed by the positions from Ile102 to Asp108 (Fig 3B and 3C) tends to become closer to the active site pocket of the other subunit while in the apo form it fluctuates on the protein surface. The C-terminal side of the linker (residues 81–100) is more constrained in holo form compared to the apo form where it oscillates on the protein surface. At the same time, the holo wHTH domain detaches from the surface of the subunit and fluctuates around the bond connecting to the linker in correspondence of the residue Leu82. Within the time span used in this work, no significant motion have been detected for the wHTH of the other subunit and, likewise, for the wHTH domains of apo form as well.

### Relevant residue interactions

The GabR structure areas most subject to dynamics movements have been delimited, as described above, by visually comparing the apo and the holo forms at the beginning and at the end of the MD simulations. The most changing regions turned out to be the linker and the interface between the wHTH and AAT domain. Molecular details of the changes taking place at these locations at the level of the composing residues have been analyzed in more details. In particular, the residue interactions have been monitored through analysis of the variations of their structural parameters *versus* simulation time. Several interactions form or break during the simulation of the GabR dynamics (Table 1) in the holo or apo forms and a few conformational changes occur which may be relevant for the protein function.

**Table 1:** Relevant side chain interactions.

		Holo	Apo
Interacting residues	Interaction type^a)^	Occurrence^b)^	Occurrence^b)^
Arg207B-Asp105B	Charge-charge	-+	-
Arg451B-Asp108B	Charge-charge	+	-
Lys453B-Asp108B	Charge-charge	+	-
Ile104B-Leu340A-Pro148A	Hydrophobic interaction	+	-
Glu101B-Arg155A	Charge-charge + H-bond	+	+-
Arg331A-Asp98B	Charge-charge + H-bond	+	+-
Leu99B-Phe93B-Pro326A	Hydrophobic interaction	+	-
Arg140A-Asp120B	Charge-charge + H-bond	+-	+
Asp144A-Arg451B	Charge-charge	+-	-+
Arg319B-Asp144A	Charge-charge	-+	+-
Arg140A –Glu455B	Charge-charge	+	-
Glu89B-Lys25B	Charge-charge	-	+
Glu88B-Arg300A-Asp83B	Charge-charge	-	-+

^a)^ A H-bond is considered as existing if it is observed for at least 50% of the simulation time by VMD analysis [60]

^b)^ Qualitative description of the occurrence of the interaction: + or − mean that the interaction is or is not observed during the simulation time, respectively. -+ or +- means that the interaction tends to form or to break at the end of simulation time, respectively. Quantitative description is reported in the figures of article text. Residues are indicated with the three-letter code followed by the sequence number and the chain identifier.

Formation and persistence (or occupancy) of salt bridges has been monitored by the VMD Salt Bridges plugin 1.1 [60] using the value 4.0 Å as the nitrogen-oxygen distance cut-off [66].

**Fig 4:**
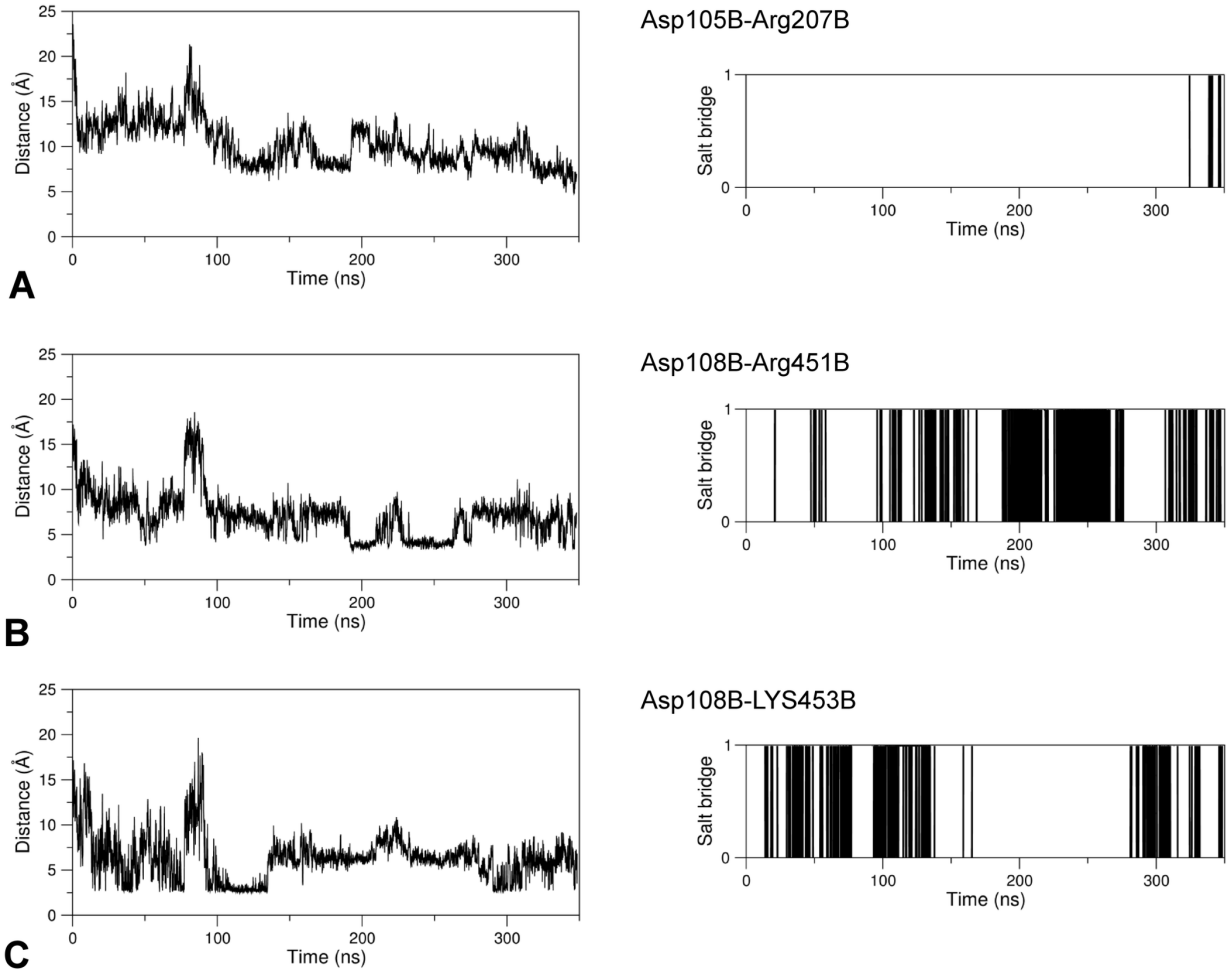
Salt bridge formation and persistence. Right and left columns of each panel report the graph of the variations of the distance between the center of mass of the oxygen atoms in the acidic side chain and the center of mass of the nitrogen atoms in the basic side chain as simulation time and the plot of existence of the corresponding salt bridge at a given time, respectively. In the latter plot, a vertical bar indicates salt bridge existence at the corresponding simulation time. The salt bridges here analyzed exist only in the GabR holo form over the simulation time. (A), (B) and (C) refer to the pairs Asp105B-Arg207B, Asp108B-Arg451B, and Asp108B-Lys453B, respectively.

At the level of the linker region delimited by the sequence positions 102–108 of the subunit B, several H-bonds, salt-bridges and hydrophobic interactions form in holo GabR with partner residues from the AAT domain. A few of these interactions are absent in the apo form (Table 1). In particular, three charge-charge interactions form in chain B between residues from the AAT domain and the linker respectively: Arg207B-Asp105B, Arg451B-Asp108B, and Lys453B-Asp108B. According to the VMD Salt Bridges plugin the interactions exist only in the holo GabR form and are absent in the apo form (Fig 4).

**Fig 5:**
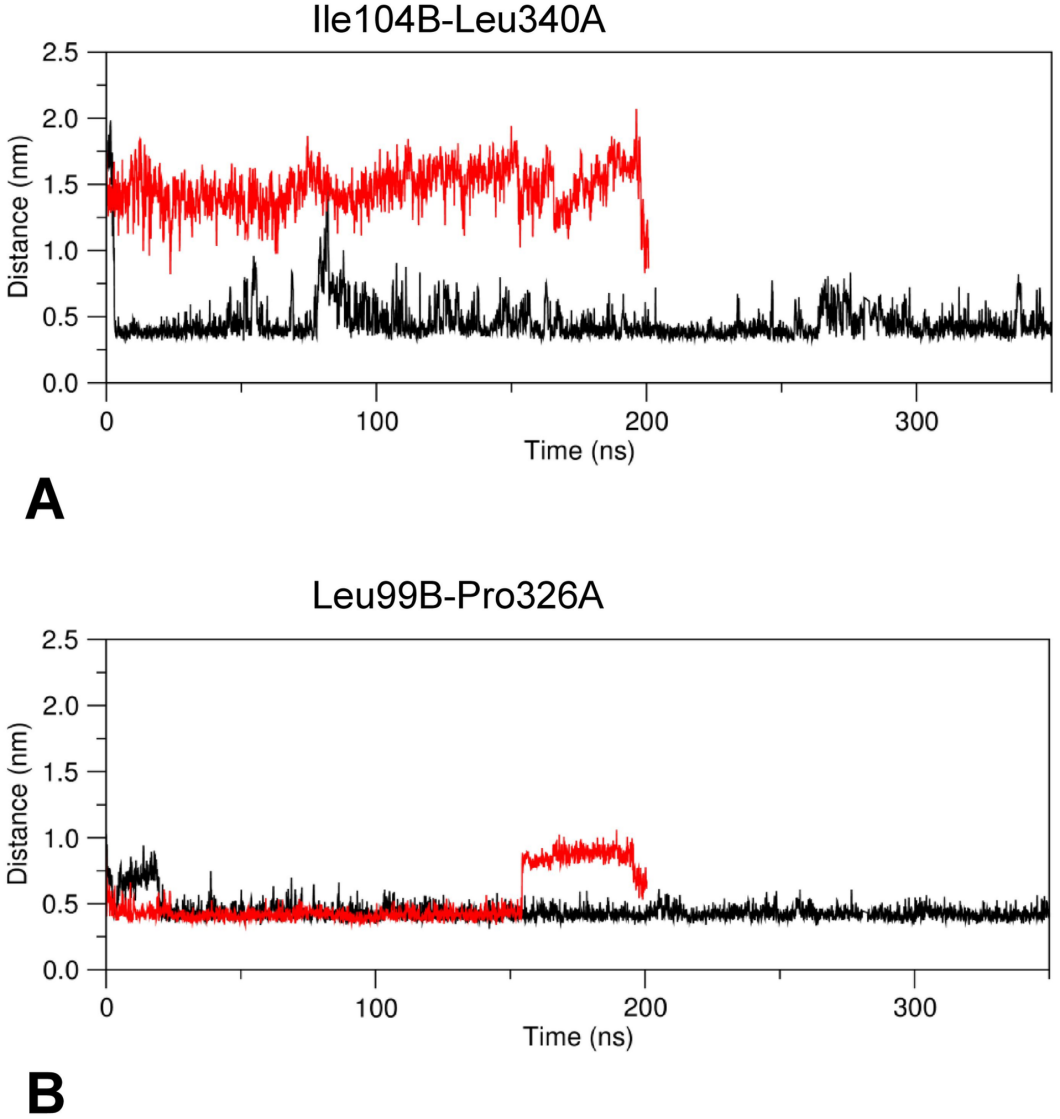
Minimum distance plot. Plot of the minimum distance between the hydrophobic residues Ile140B and Leu340A (A) and Leu99B and Pro326A (B) versus simulation time. Black and red line traces refer to holo and apo GabR, respectively.

These salt bridges are rather persistent during dynamics (Fig 4) with the exception of the ion pair Asp105B-Arg207B that tends to form only at longer simulation times (Fig 4A). Interestingly, Arg207 is conserved among GabR homologs and is structurally equivalent to the Arg192 which in GABA aminotransferase interacts with the substrate (or substrate analog) carboxylic group [25, 32, 67]. A hydrophobic interaction takes place between the linker Ile104B and Pro148A, Leu340A of the AAT domain forming a hydrophobic cluster. All these interactions are not observed in the apo form (Fig 5A).

**Fig 6:**
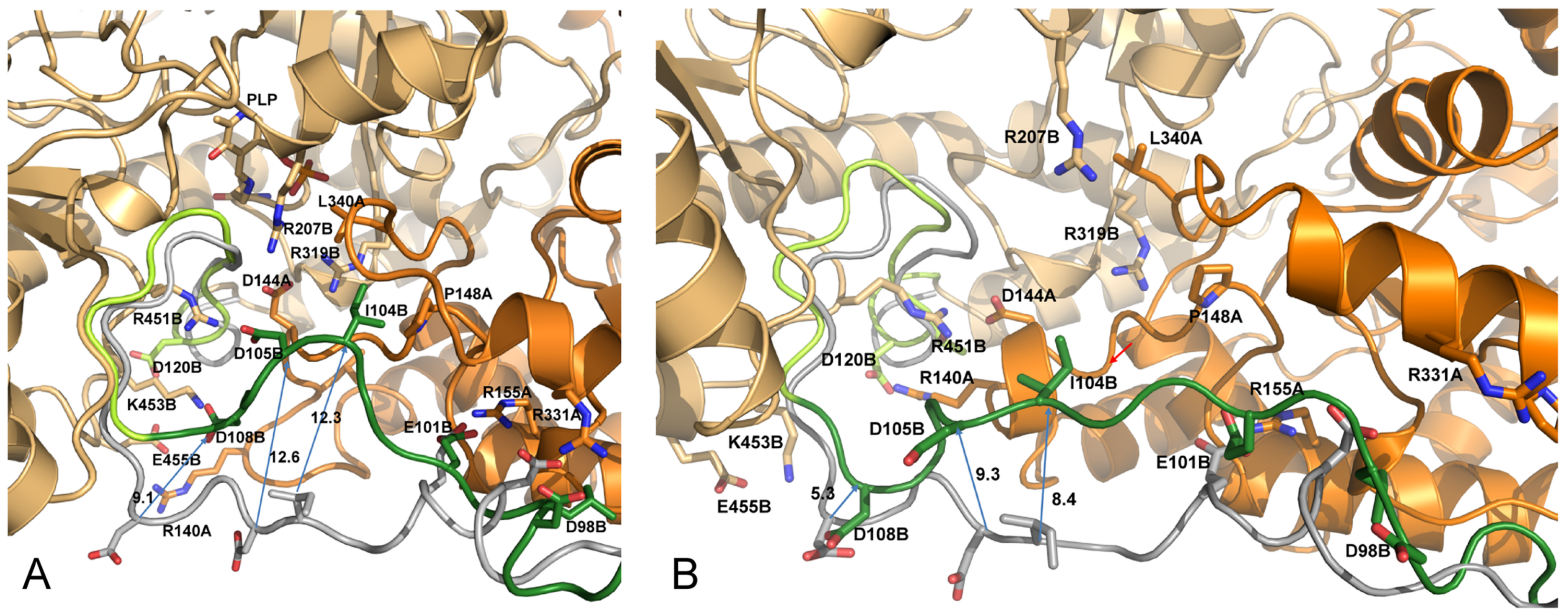
Linker-AAT domain interactions. Interactions between the linker and the AAT domain of the (A) holo and (B) apo GabR dimer in the last MD frame are displayed. Dark and light orange distinguish the two subunits represented as cartoon models. Green and grey ribbon corresponds to the linker region at the end and at 0 ns of molecular dynamics respectively. The yellow ribbon segment in the last frame structures indicate the loop connecting the linker to the first N-terminal helix of the AAT domain. Relevant side chains are displayed with stick models. Residues are labeled with the one-letter code followed by sequence positions and chain id. Arrows connect positions of equivalent residues at the start and end of MD simulation. Decimal figures associated to the arrows indicate the approximate distance between the Cα carbons of the connected residues. Red arrow in (B) points to the one-turn helix.

All these interactions tend to form during MD simulation in the holo form (Fig 6A) while are missing in the apo form throughout all the simulation time (Fig 6B).

**Fig 7:**
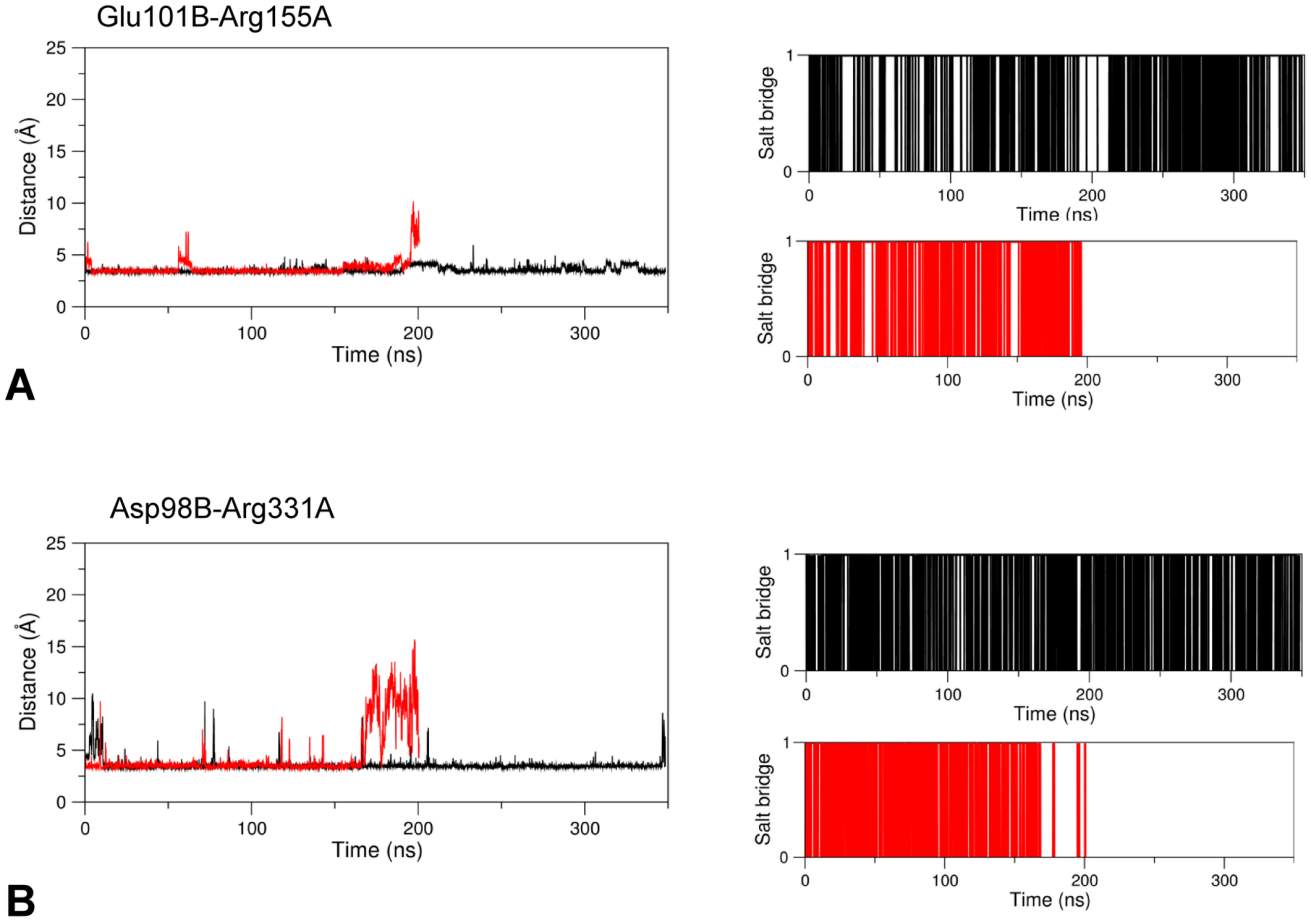
Salt bridge formation and persistence. Right and left plots of each panel are as in Fig 4. Black and red lines refer to the holo and apo GabR forms, respectively. The salt bridges here analyzed are (A) Glu101B-Arg155A and (B) Asp98B-Arg331A.

In the linker region encompassed by sequence positions 84–101, the interactions between Arg155A and Glu101B (in the linker) and Arg331A and within linker Asp98B, occurring in the holo form, weaken in the apo form only at the end of the simulation time with increasing average distances between side chains (Fig 7).

**Fig 8:**
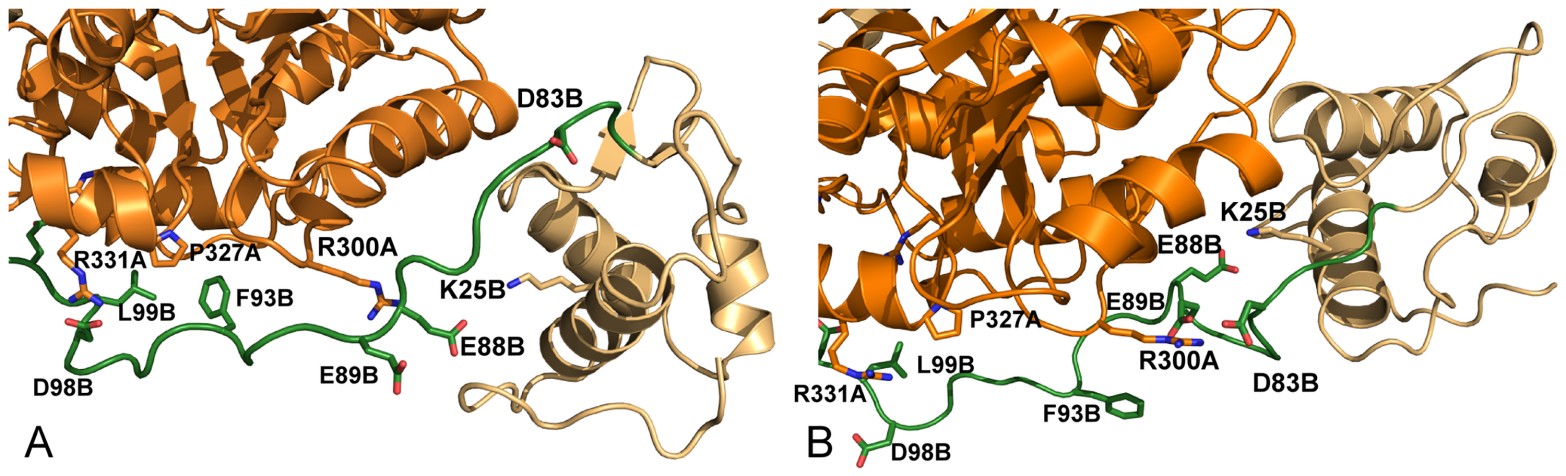
Interactions in proximity of the wHTH domain. Colors, model representations and residue labels are as described in Fig 6 caption except for the linker at 0 ns, not reported here.

According to these observations, the linker tract containing the residues Asp98 and Glu101 may be considered a sort of hinge around which the linker movement takes place. The hinge is reinforced by the formation of a hydrophobic cluster involving the linker residues Leu99B, Phe93B and Pro326A from AAT domain of subunit A (Figs 5B and 8).

**Fig 9:**
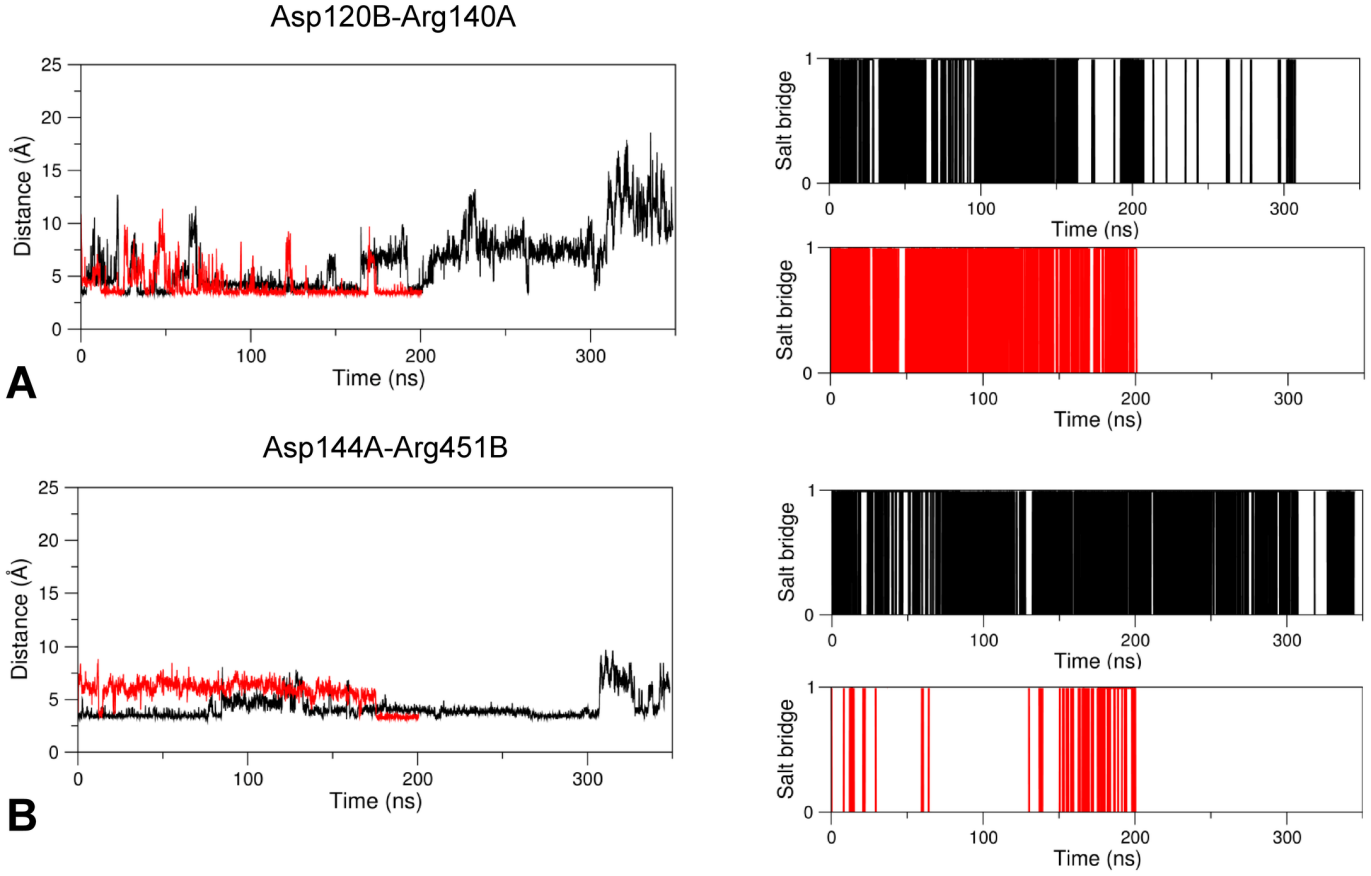
Salt bridge formation and persistence. Plots are interpreted as in Fig 4. Salt bridges analyzed are (A) Asp120B-Arg144A and (B) Asp144A-Arg451B. Black and red lines refer to holo and apo GabR forms, respectively.

Other interesting interactions not involving directly the linker region but potentially relevant to the conformational transitions of GabR could be observed. In the apo form, the salt bridges Arg140A-Asp120B and Asp144A-Arg451B cooperate to the stabilization of the one-helix turn stretch delimited by the residues 140A-144A (Figs 6 and 9). Asp120B is located in the loop that connects the linker to the first N-terminal helix of the AAT domain. This short helix hinders entrance of the linker in the cavity near the active site.

**Fig 10:**
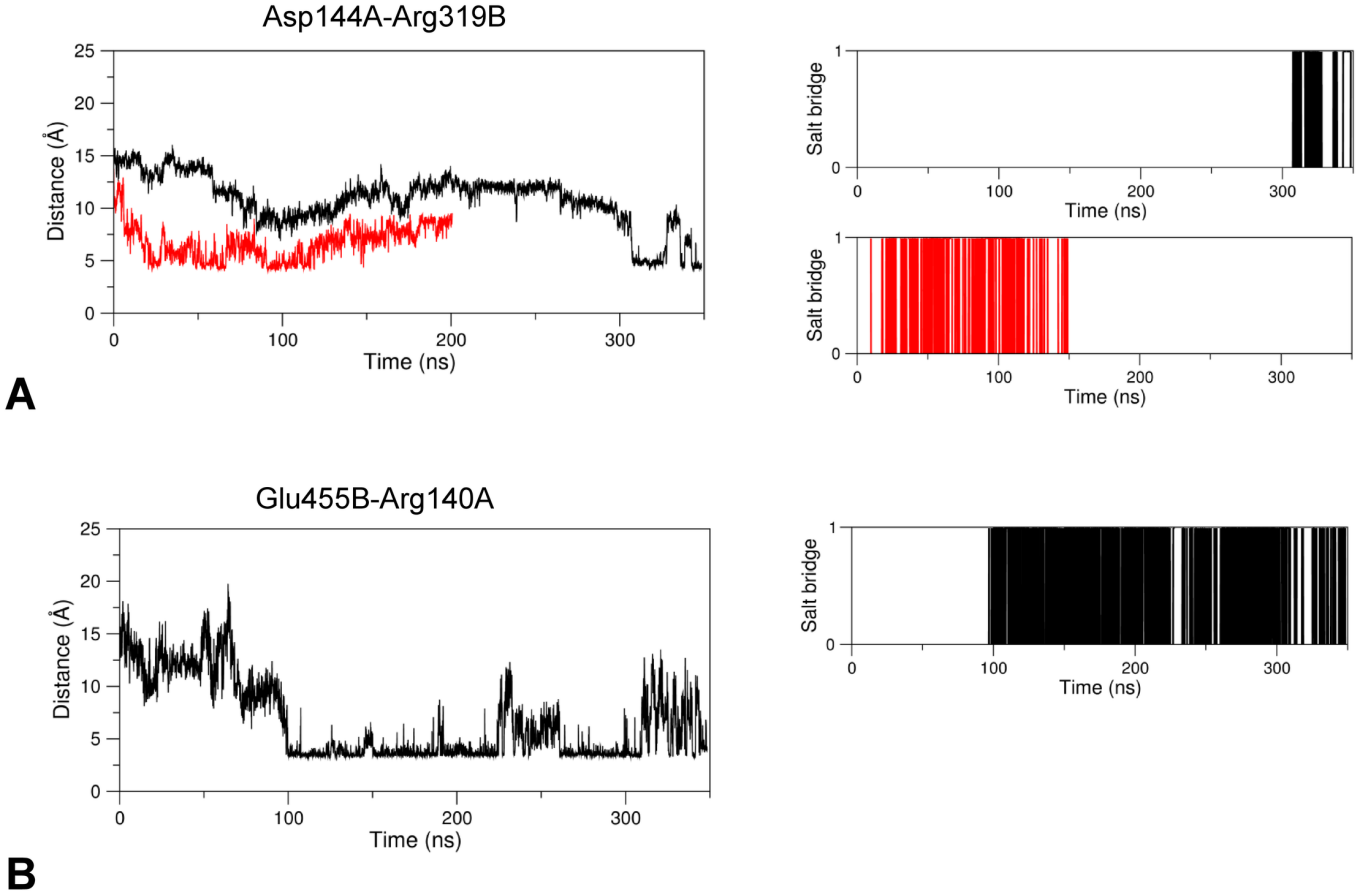
Salt bridge formation and persistence. Plots are interpreted as in Fig 4. Salt bridges analyzed are (A) Asp144A-Arg319B and (B) Glu455B-Arg140A, present only in holo GabR. Black and red lines refer to holo and apo GabR forms, respectively.

In the holo form, these residues rearrange their interactions: Asp144A interacts with Arg319B which, in the apo form, points to a direction opposite to the PLP phosphate binding site at 7.0 Å from the carboxylic oxygen of Asp144A (Fig 6); Arg140A interacts with Glu455B. The conformational modifications of Arg140A and Asp144A are reflected by the increase in the distances to their partners in the late frames of the holo form dynamics (Figs 9 and 10). These two new interactions in the holo GabR form promote the local unfolding of the one-turn helix at positions 140A-144A and the removal of the obstacle to the access to the cavity which facilitates the docking of the linker toward the active site.

It can be speculated that the presence of the negatively charged phosphate group at the active site induces a local conformational reconfiguration of GabR main and side chains which triggers the unfolding of the one–helix loop. The change of local conformation opens access to the active site pocket and allows docking of the linker with consequent induction of the movement of the corresponding wHTH domain. The mediators of such an effect appear to be Arg319B and Arg207B that, in presence of the PLP bound, undergo a conformational change of their side chains that stabilizes charge-charge interaction with Asp144A and Asp105B, respectively. Plots reporting the RMSD variations over the initial conformations of the side chains of Arg319B and Arg207B versus simulation time suggest that, in holo GabR, these residues undergo a rearrangement of their side chains (S3 Fig). Interestingly, as Arg207B, also Arg319 is conserved among GabR homologs.

**Fig 11:**
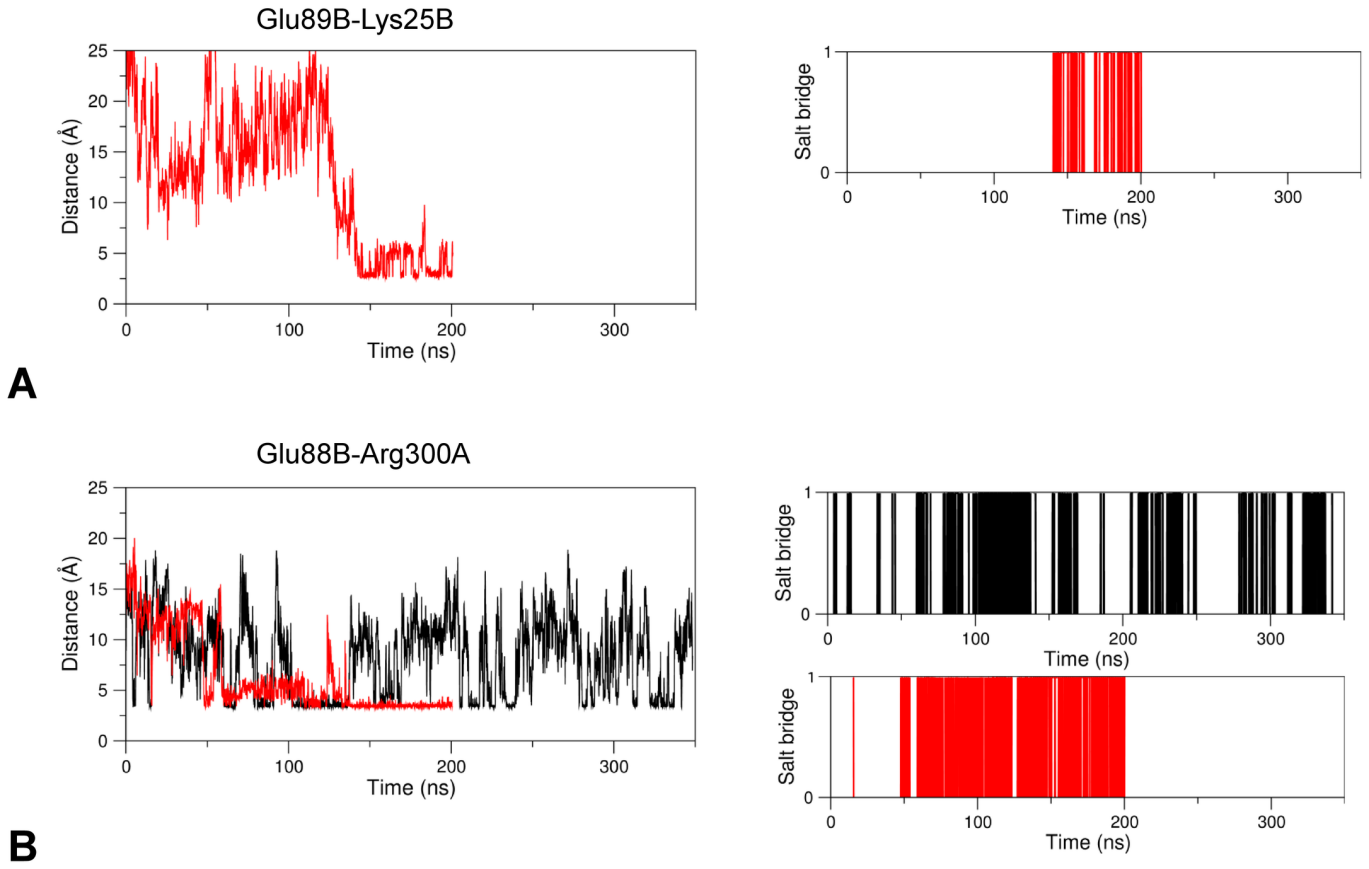
Salt bridge formation and persistence. Plots are interpreted as in Fig 4. Salt bridges analyzed are (A) Glu89B-Lys25B, only in apo GabR and (B) Glu88B-Arg300A. Black and red lines refer to holo and apo GabR forms, respectively.

Other notable interactions involving the linker are Glu89B-Lys25B, that connect the linker to the wHTH domain, and Glu88B-Arg300A-Asp83B, which bridges the linker and the AAT domain of the other subunit. These interactions are taking place in the apo form of GabR where they contribute to anchoring the wHTH domain to the surface of the AAT subunit; they are missing in the holo form (Fig 7) where distances between charged groups tend to increase with time (Fig 11).

**Fig 12:**
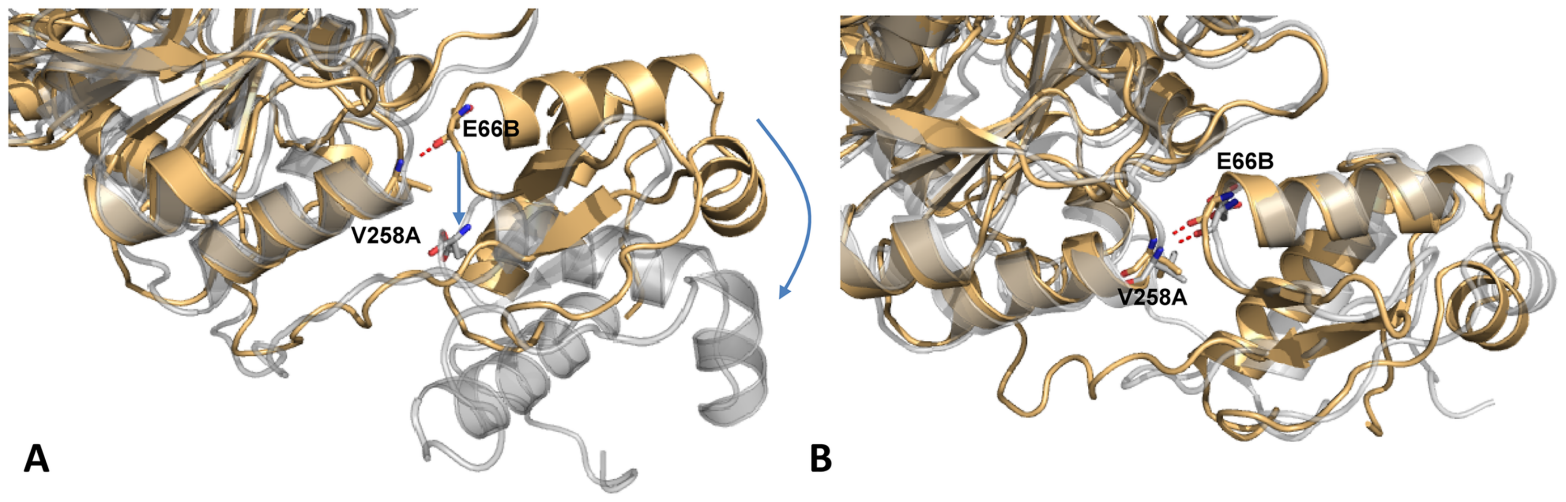
Comparison between the initial and final MD frames of holo and apo GabR. The initial (orange) and final frames (transparent grey) of holo (A) and (B) apo GabR are displayed in correspondence of the interaction between the wHTH and the AAT domain of the other subunit. The H-bond connecting the wHTH helix Val53-Glu66 to the AAT helix Val258-Ala268 is shown as a red dashed line. Arrows indicate the position of the wHTH residue and helix in the last MD frame. Residues are labeled as in Fig 6.

During the movement of the wHTH domain in the holo GabR, a H-bond connecting the carbonyl oxygen of the peptide bond at Glu66B to the peptide bond nitrogen of Val258A of the other chain, breaks. The two residues belong to two α-helices (Val53-Glu66 of the wHTH and Val258-Ala268 of the AAT domains, respectively) which, in the initial structure, are aligned through their axes (Fig 12A) forming a virtual, single long α-helix. The same H-bond does not break in the GabR apo form (Fig 12B) and contributes to keep the two helices close to each other and aligned.

## Discussion

This work was conceived to contribute to the delineation of the potential conformational changes in the transcriptional regulator GabR from *B*. *subtilis* using a molecular dynamics approach.

Only the structures of GabR without any ligand, complexed with imidazole or pyridoxal 5’-phosphate bound as internal aldimine were available at the beginning of this work. Ideally, the complex with PLP and the effector GABA (or one of its analogs) bound as external aldimine would be necessary for a complete description of the system in its active form and to understand the basis for repressor activity. Moreover, the availability of the structure of a complex with the target DNA would provide an exhaustive picture of the system at work. Therefore, only the experimentally determined apo and holo (PLP bound) forms were used to simulate their motion and the results were compared to highlight differences possibly related to the presence of PLP. Apo GabR had in the context of this work the role of the reference structure against which to compare the holo form dynamics and highlight conformational changes related to the presence of PLP at the active site. Although the apo and PLP-binding forms have been proved to be able to bind DNA but not to trigger gene transcription, the differences in the dynamics behavior of the two states may be indicative of the intramolecular movements taking place in the fully functional GabR bound to PLP and GABA [13, 25]. Recently, the crystallographic structures of two forms of the AAT-like domains of GabR in complex with PLP and GABA have been deposited in the Protein Data Bank [26, 27]. The availability of these structures paves the way to the analysis of the interactions taking place at the active site in the presence of the effector GABA. The transition from the open to closed form upon effector binding, typical of the fold type-I PLP dependent enzymes, has been observed in the AAT domain dimer deposited as PDB code 5X03. This transition is very likely one of the keys to understand the action of the effector. Unfortunately, all these complexes have been crystallized without the wHTH domains and their linkers: therefore an important component of the system is still lacking. Nonetheless, these structures represent a valuable starting point for further investigations.

Comparison of the molecular dynamics behavior of the holo and apo GabR forms showed that the most evident difference is the conformational change in the holo PLP-bound form represented by the rotation and the subsequent detachment from the subunit surface of one of the wHTH domains. Apparently, the movement is mediated, and maybe caused, by a rearrangement of the linker connecting the AAT domain. This is the second most “macroscopic” conformational modification. The C-terminal section of the linker docks into the “active site” pocket and establish stabilizing contacts consisting of hydrogen-bonds, salt-bridges and hydrophobic interactions (Table 1 and Fig 6). The N-terminal moiety of the linker, the part immediately connected to the moving wHTH domain, tends to move farther from the subunit surface and, during this movement, at least three salt-bridges are broken. None of these changes can be seen in the apo form during the simulation time span. Rebuilding of the two short loops in the holo form, not needed in the apo form, is very unlikely to have introduced any artifact in the dynamics because the two loops are located in a solvent exposed position of the AAT domains not interacting with linker or wHTH domain surfaces (Fig 1). All these considerations emphasize the importance of the linker as an essential component of the molecular mechanism of the transcriptional regulator response to effector binding.

How could the conformational transitions taking place in the holo form be explained? Analysis of the trajectories points to a rearrangement of charged side chains present at or in proximity of the active site, probably triggered by the presence of the negatively charged PLP phosphate group. Arg207B and Arg319B may function as “sensors” of presence of negative charges at the active site. In particular, Arg207 is equivalent to the Arg192 that in GABA aminotransferase is supposed to interact with the substrate carboxylic group of γ-amino butyrate and it is conceivable that Arg207 plays a similar function in GabR. Indeed, the crystallographic structure of the GABA bound AAT domain of GabR corroborates this conclusion. In presence of PLP, Arg207B side chain looses flexibility at variance with what happens in apo GabR and the resulting stiffness contributes to open the active site cavity. Consequently, the linker can dock to the active site and the linker residue Asp105B can interact with nearby positively charged side chains to stabilize the contact. Similar considerations can be drawn for Arg319B whose side chain is attracted by the active site negative charge and is oriented in such a way to interact with Asp144A. This interaction contributes to open the access to the active site cavity. According to this model, the effect of the phosphate group of PLP is reminiscent of the allosteric transitions induced by residue phosphorylation [68].

**Fig 13:**
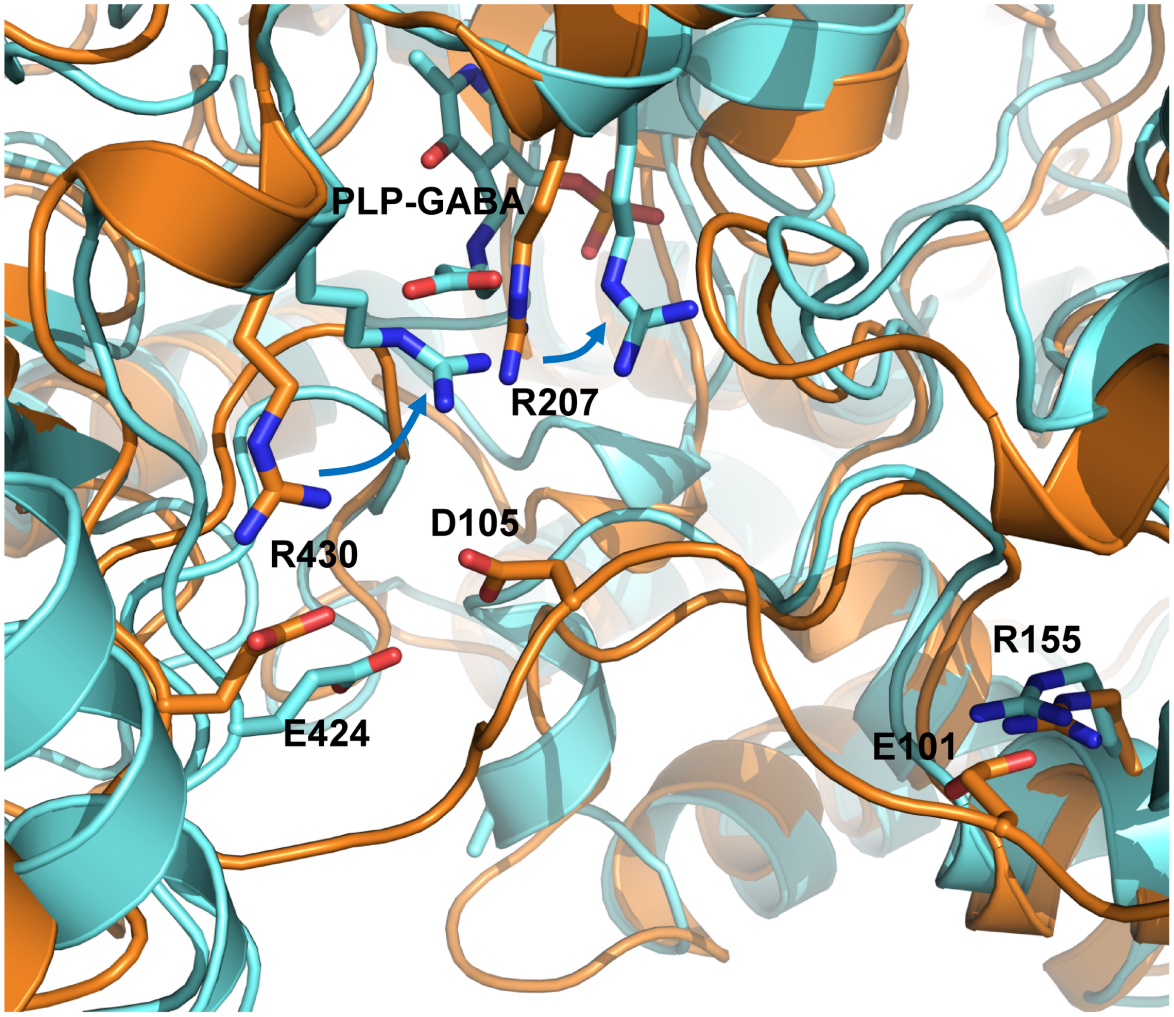
Structural superposition between the AAT-like domain subunit B of 5T4J (cyan ribbon) and the last frame of MD simulation of holo GabR (orange). Relevant side chain are displayed as stick models and labeled with one letter code. Blue arrows indicate the correspondence between the discussed Arg residues in the two structures.

However, the MD simulation of holo GabR has been carried out without the physiological effector γ-amino butyrate, which is able to activate the GabR mediated transcriptional activation. It can be speculated that the presence of the negatively charged carboxylic group of the GABA at the active site of the holo GabR, in addition to that of the phosphate group, would enhance the attraction of the linker and the movement of the attached wHTH domain. In an attempt to support this hypothesis, the structural superposition between the AAT domain complexed with the PLP-GABA external aldimine from the PDB structure 5T4J and the last frame of the MD simulation of holo GabR form has been examined (Fig 13). The crystallographic structure shows that at the active site, the carboxylic group of GABA interacts with Arg207 and Arg430, both conserved among homologous GabRs. The presence of the GABA carboxylic group attracts toward the active site these two positively charged side chains, in particular Arg430 which otherwise would be involved in a salt bridge with Glu424 (Fig 13). This rearrangement creates a cluster of positive charges and delineates a cavity in which the Asp105 of the linker could be pulled by electrostatic forces.

In addition to that, the binding of the physiological ligand induces, as demonstrated by the crystallographic experiments, the transition from the open to closed conformation of GabR which may significantly contribute to boost the rearrangement of the linker and the wHTH movement and detachment. Therefore, it can be envisaged that the herein reported molecular dynamics studies were able to observe the detachment of only one of the two domain because of one, or a combination of, the following possible reasons: the simulation time period is not long enough for the detachment of the other wHTH domain to occur; all the required interactions do not form at the active sites cause of the absence of the full complex with the physiological effector GABA; the possible existence of allosteric interferences between the two sites as suggested by the authors of the structure 5x03 [27].

In conclusion, this work has proposed an hypothetical model describing the potential structural rearrangements of GabR upon PLP binding. The observed modifications can suggest the possible changes taking place in the regulator upon GABA binding and external aldimine formation. The model proposes a view implying that formation of the PLP-GABA adduct at GabR active site triggers a rearrangement of the linker that is attracted toward the active site of the other subunit by means of electrostatic forces. Residues mainly involved in this transition are linker Asp105, which is the negative charge attracted by Arg207 and Arg430, and Glu101 that forms the linker hinge through interaction with Arg155. Conformational rearrangement of the linker, in synergy with the open to close transition, causes detachment of the wHTH domains. The detachment of the wHTH domains would modify interaction of GabR with DNA and possibly recruit DNA polymerase and other factors for transcription. This picture is compatible with the models already published in the literature whose shared conclusion is that GabR undergoes a conformational transition upon GABA binding [25–27, 33, 35]

Even if the model proposed has the limitation of having been derived without the effector GABA bound at GabR active, it can provide a general and testable framework for the interpretation of the action of the regulators of the MocR subfamily. Indeed, the model indicates a set of residues potentially involved in the response to ligand binding the role of which can be tested by site-directed mutagenesis.

## Supporting information

S1 Fig RMSD and RMSF variations of GabR with and without imidazole. RMSD and RMSF are reported in (A) and (B), respectively. Color code is indicated in the inset of the plot. Calculations have been carried out taking into account only the main chain atoms.
(PDF)

S2 Fig RMSD and RMSF variations of the wHTH domains of holo and apo GabR. RMSD and RMSF are reported in (A) and (B), respectively. Color code is indicated in the insets of the plots. Calculations have been carried out taking into account only the main chain atoms.
(PDF)

S3 Fig RMSD variations. Plot of the RMSD over the initial conformation of the residues Arg319B (A) and Arg207B (B).versus simulation time. Black and red line traces refer to holo and apo GabR, respectively.
(PDF)

S1 File PDB coordinates of the holo GabR form with hydrogen added, used for molecular dynamics simulation. (PDB)

S2 File PDB coordinates of the apo GabR form with hydrogen added and rebuilt loops, used for molecular dynamics simulation. (PDB)

S1 Movie Movie of the 350 ns simulation of the holo GabR form. PLP is represented by space filling models. Movie file is encoded with H.264 in an MP4 container.
(MP4)

S2 Movie Movie of the 200 ns simulation of apo GabR. File format is as in S6 Movie.
(MP4)
