# Supplementary material for: Molecular dynamics simulation unveils the conformational flexibility of the interdomain linker in the bacterial transcriptional regulator GabR from Bacillus subtilis bound to pyridoxal 5’-phosphate
Source: PLoS One. 2017 Dec 18;12(12):e0189270. doi: 10.1371/journal.pone.0189270 (PMC5734734; doi:10.1371/journal.pone.0189270)
Supplement: S1 Fig — RMSD and RMSF are reported in (A) and (B), respectively. Color code is indicated in the inset of the plot. Calculations have been carried out taking into account only the main chain atoms. (PDF) [file pone.0189270.s001.pdf]

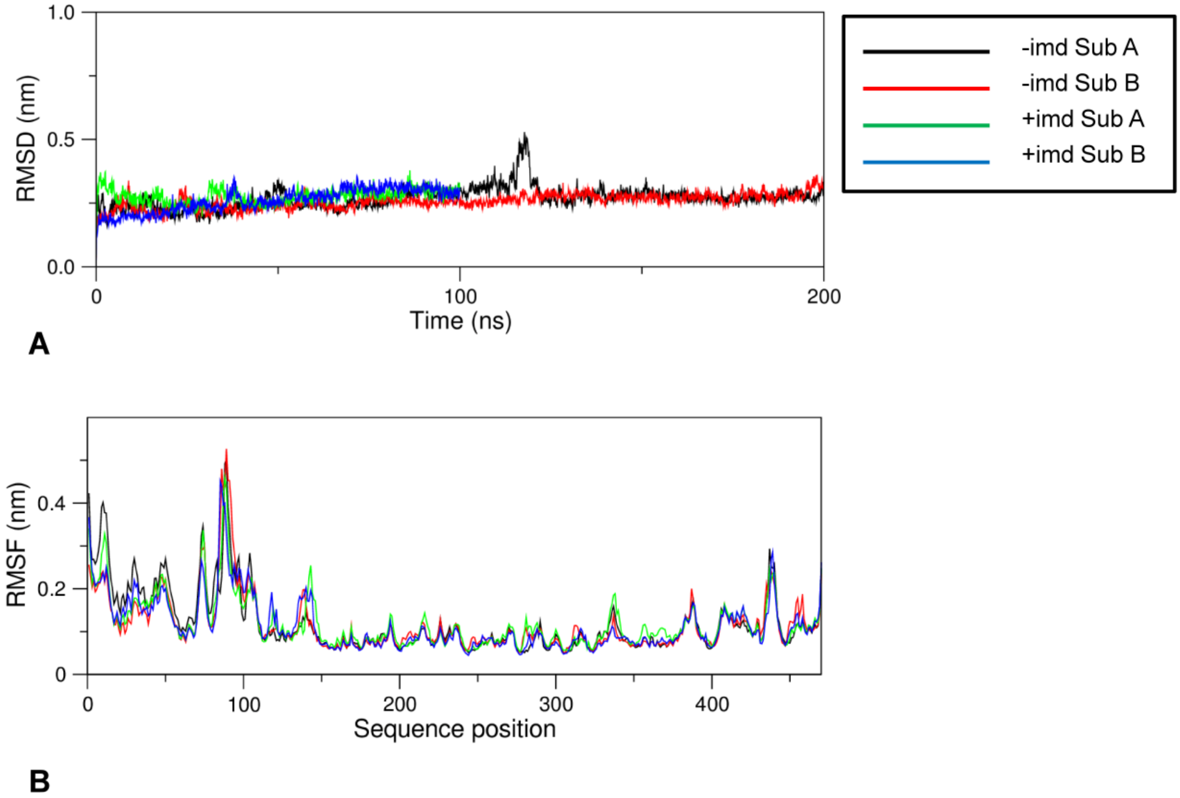

S1 Fig. RMSD and RMSF variations of GabR with and without imidazole. RMSD and RMSF are reported in (A) and (B), respectively. Color code is indicated in the inset of the plot. Calculations have been carried out taking into account only the main chain atoms.
