# Supplementary material for: Molecular dynamics simulation unveils the conformational flexibility of the interdomain linker in the bacterial transcriptional regulator GabR from Bacillus subtilis bound to pyridoxal 5’-phosphate
Source: PLoS One. 2017 Dec 18;12(12):e0189270. doi: 10.1371/journal.pone.0189270 (PMC5734734; doi:10.1371/journal.pone.0189270)
Supplement: S3 Fig — Plot of the RMSD over the initial conformation of the residues Arg319B (A) and Arg207B (B).versus simulation time. Black and red line traces refer to holo and apo GabR, respectively. (PDF) [file pone.0189270.s003.pdf]

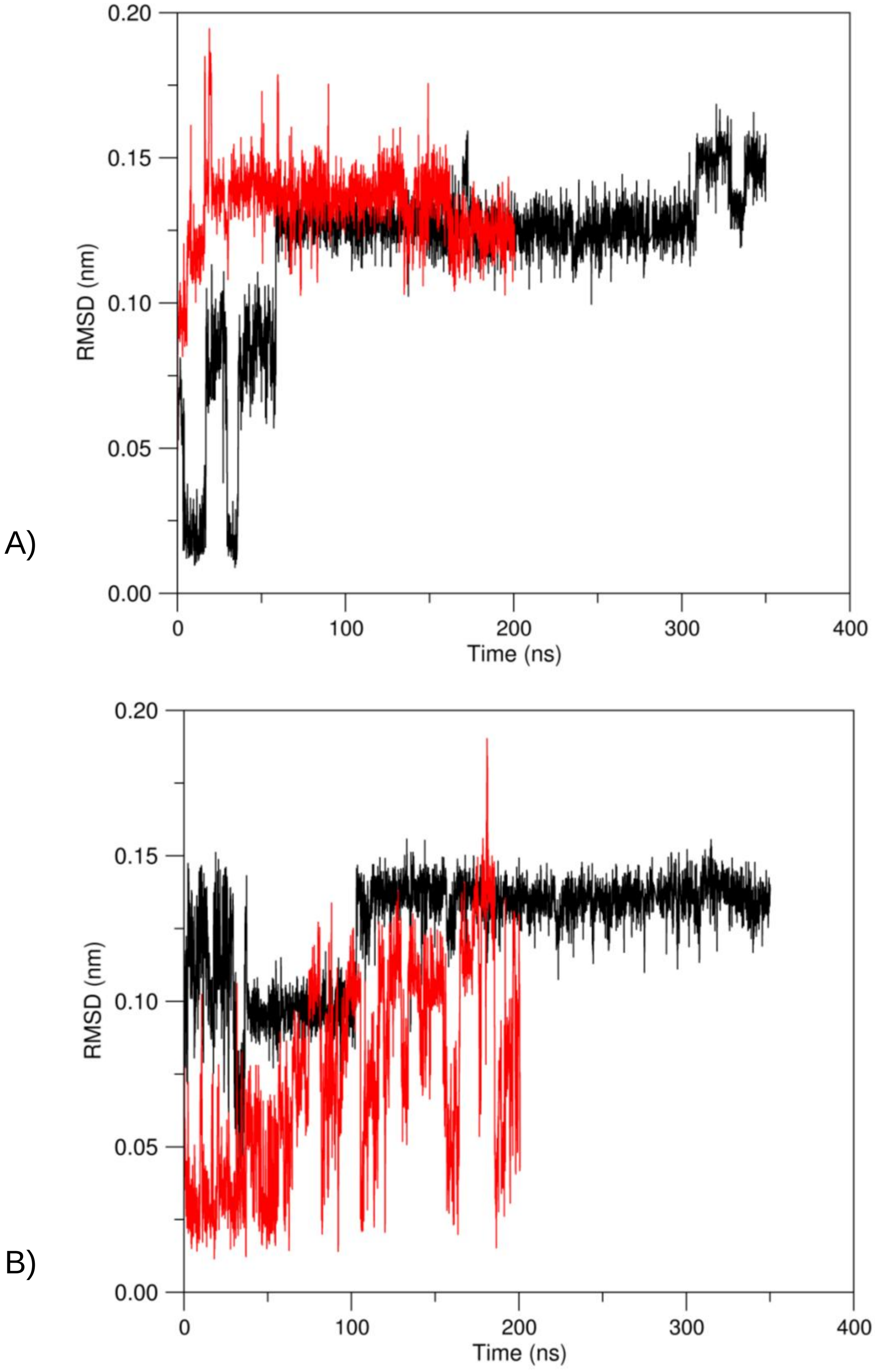

S3 Fig. RMSD variations. Plot of the RMSD over the initial conformation of the residues Arg319B (A) and Arg207B (B).versus simulation time. Black and red line traces refer to holo and apo GabR, respectively.
